# Supplementary material for: Inducible chromatin priming is associated with the establishment of immunological memory in T cells
Source: EMBO J. 2016 Jan 21;35(5):515–35. doi: 10.15252/embj.201592534 (PMC4772849; doi:10.15252/embj.201592534)
Supplement: Supplementary file 1 — Appendix [file EMBJ-35-515-s001.pdf]

APPENDIX

Supplementary figures and methods

Inducible chromatin priming is associated with the establishment of immunological memory in T cells.

Sarah L. Bevington, Pierre Cauchy, Jason Piper, Elisabeth Bertrand, Naveen Lalli, Rebecca C. Jarvis , L. Niall Gilding , Sascha Ott, Constanze Bonifer, and Peter N. Cockerill

Data sets accompanying this manuscript:

Dataset EV1 – 2882 primed DHSs, accompanying Figure 3A.

Dataset EV2 – 1895 inducible genes, accompanying Figure 3F.

Dataset EV3 – 683 inducible genes, accompanying Figure 3G.

Dataset EV4 – 1217 inducible DHSs, accompanying Figure 4A.

Dataset EV5 – 10 motifs identified in pDHSs or iDHSs, accompanying Figures 9A and 9B.

Supplementary Figure Legends

Appendix Figure S1

Properties of $CD4^+$ naïve, blast and memory-phenotype T cells.

(A) Human *CSF2*, mouse *Ccl1* and mouse *Fos* mRNA expression in $CD4^+$ T_N stimulated with Concanavalin A for the times indicated. The level of mRNA is normalized to Beta-2-microglubulin mRNA (B2M).

(B) Representative FACS plots of $CD4^+$ T_N, $CD4^+$ T_M and $CD4^+$ T_B cells stained with antibodies to distinguish the different cell types. Cells were labeled with CD4, B220, CD44, CD62L, CD3 and CD25.

Appendix Figure S2

UCSC browser screen shot of all of the mouse genome-wide data sets defined in this study.

This window spans the Th2 cytokine gene locus which includes the *Il4*, *Il13* and *Il5* genes, and includes many other more widely expressed genes. This view shows the scales that are used in each of the other screen shots depicted in this study. The $CD4^+$ T_N H3K27ac and the three H3K27me3 tracks are from published data sets. Shown underneath are Venn diagrams showing the overlaps between the major DHS peaks identified for the replicates of the DNAse-Seq analyses of $CD4^+$ T_N, $CD4^+$ T_{N+} and $CD4^+$ T_B.

Appendix Figure S3

Influence of primed DHSs on basal and inducible levels of gene expression.

(A and B) pDHSs do not activate basal level transcription. These plots depict the average log2 mRNA array values for $CD4^+$ Memory T cells compared to $CD4^+$ naïve T cells in the absence of stimulation. Data are shown for the genes closest to the 2882 genes defined as primed DHSs (panel A) and for the 1893 genes defined as being at least 2-fold induced specifically in $CD4^+$ memory T cells (panel B).

(C) Memory T cell-specific inducible genes have pDHSs in close proximity to iDHSs. Shown here are the log2 fold increases in mRNA expression detected in response to stimulation for loci that have iDHSs located within 150 kb of a promoter of an inducible TM-specific gene. The mRNA data is plotted versus the distances between the iDHS and the nearest pDHS.

Appendix Figure S4

Overlaps for TF ChIP peaks with pDHSs and iDHSs.

Venn diagram overlaps of ETS-1, RUNX1 and JUNB ChIP-Seq peaks with the defined subsets of 2882 pDHSs and 1217 iDHSs. ETS-1 peaks from T_N and T_B cells (top); RUNX1 peaks from T_N, T_B and T_B+ cells (bottom left); JUNB peaks from T_B and T_B+ cells (bottom right).

Appendix Figure S5

Suppression of inducible gene expression in memory-phenotype cells by an inhibitor of RUNX1 function.

(A) Flow cytometric analyses of T_B prepared from $CD4^+$ T cells cultured with 2 μg/ml ConA in the presence or absence of 20 μM Ro5-3335 for 40 hours followed by 24 hours in 50 U/ml IL-2 in the presence or absence of 20 μM Ro5-3335. Cells were labeled with FITC- Annexin V and PI. A representative FACS plot from 3 experiments is shown.

(B) mRNA expression analyses of inducible gene expression in T_B prepared in the presence and absence of the inhibitor Ro5-3335 at 20 μM. Cells prepared as in (A) were left unstimulated or stimulated with PMA/I for 2 hours. mRNA expression values were normalized to the levels of *B2m*.

Appendix Figure S6

Composition of pDHSs and iDHSs

(A) T_M-specific regulation of *Syne3* Left: Upper: UCSC genome browser shot of the *Syne3* locus showing a pDHS (black arrow) and an iDHS (red arrow). Numbers represent the distance in kilobases from the *Syne3* TSS. Lower: Log2 mRNA expression levels of *Syne3* in untreated and PMA/I treated T_N and T_M taken from the microarray analyses. Right: DNA sequences of the +9 kb pDHS and the +6.5 kb iDHS. TF binding sites are underlined. The pDHS has a 3:1 ratio of constitutive to inducible TF binding sites and the iDHS a 2:1 ratio of inducible to constitutive TF sites.

(B, C) DNA sequences of the *Rad50* (B) and *Cxcr3* (C) pDHSs and iDHSs. TF binding sites are underlined.

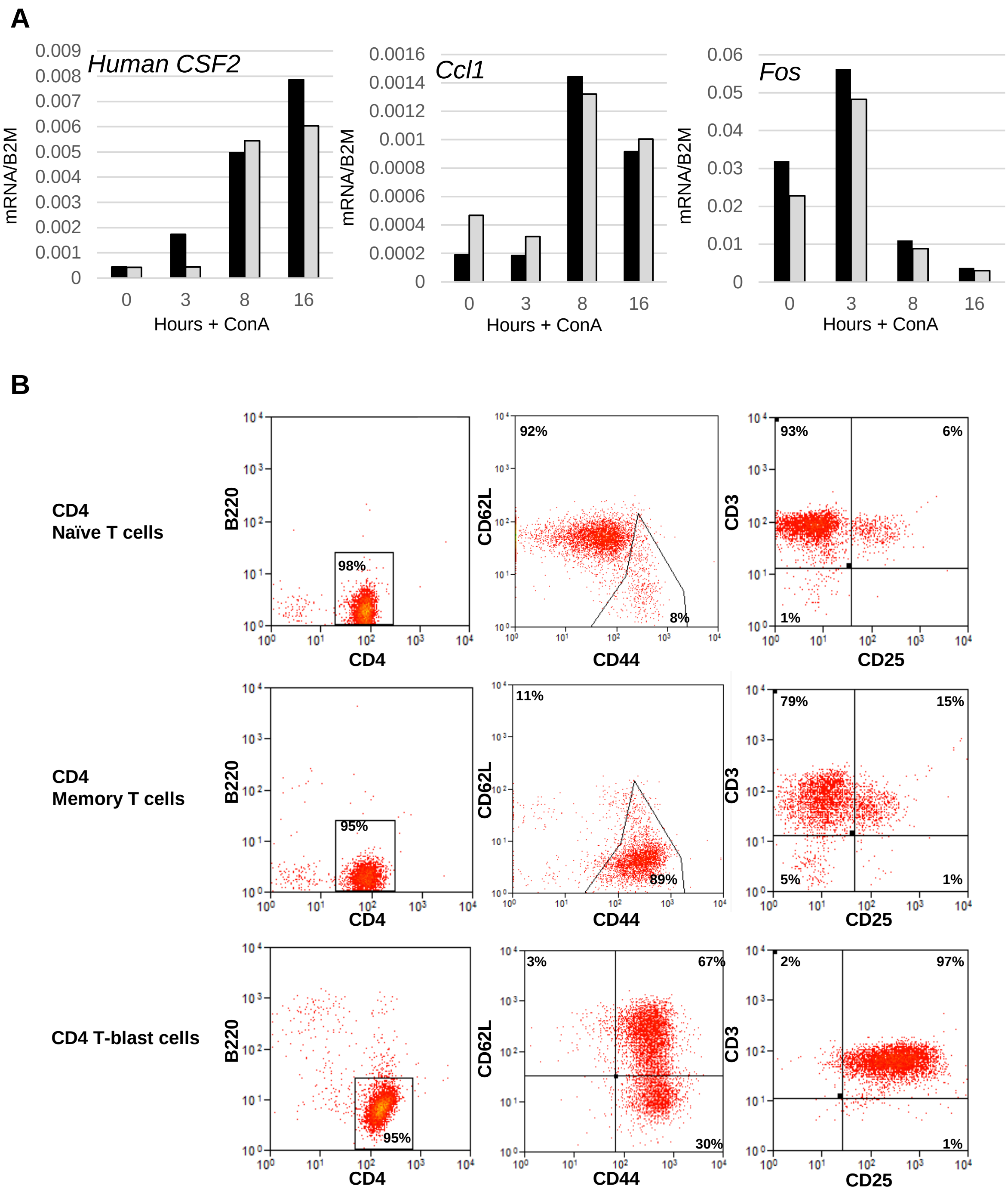

Appendix Figure S1

Properties of CD4+ naïve, blast and memory-phenotype T cells.

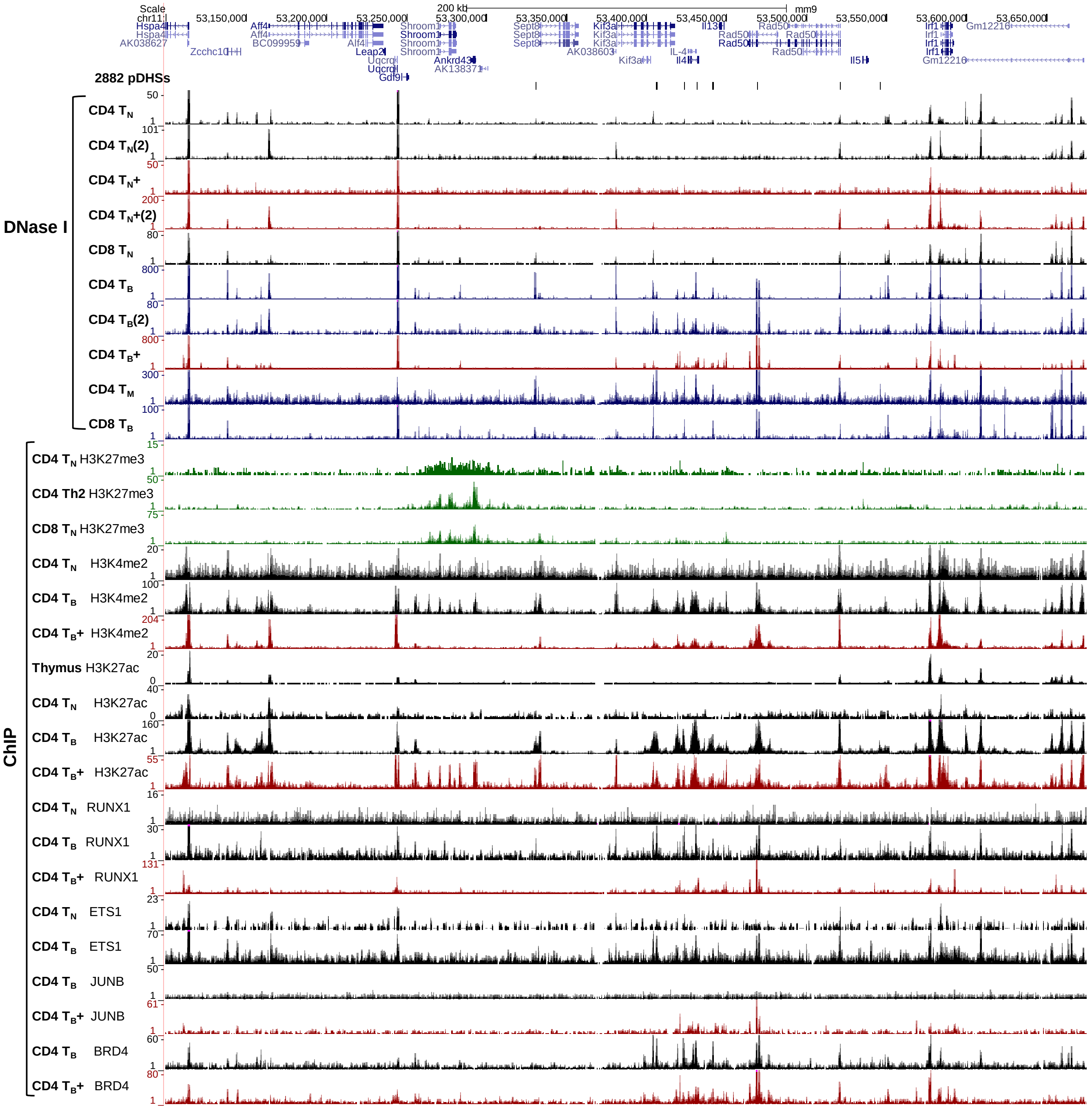

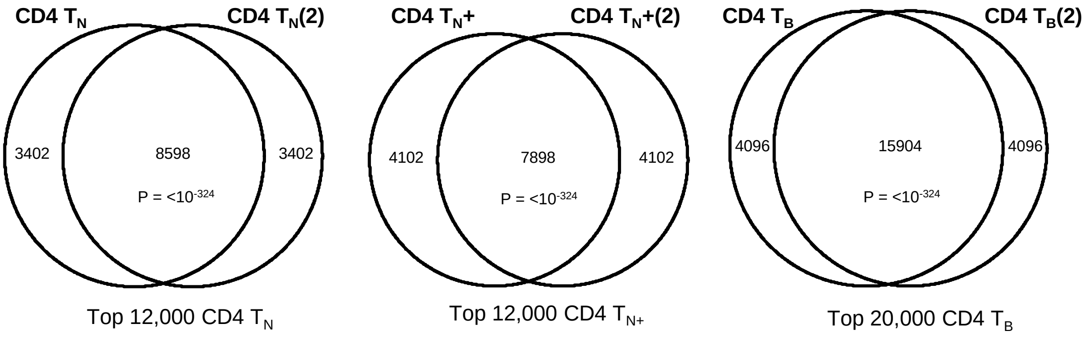

Appendix Figure S2 **UCSC browser screen shot of all of the mouse genome-wide data sets defined in this study.**

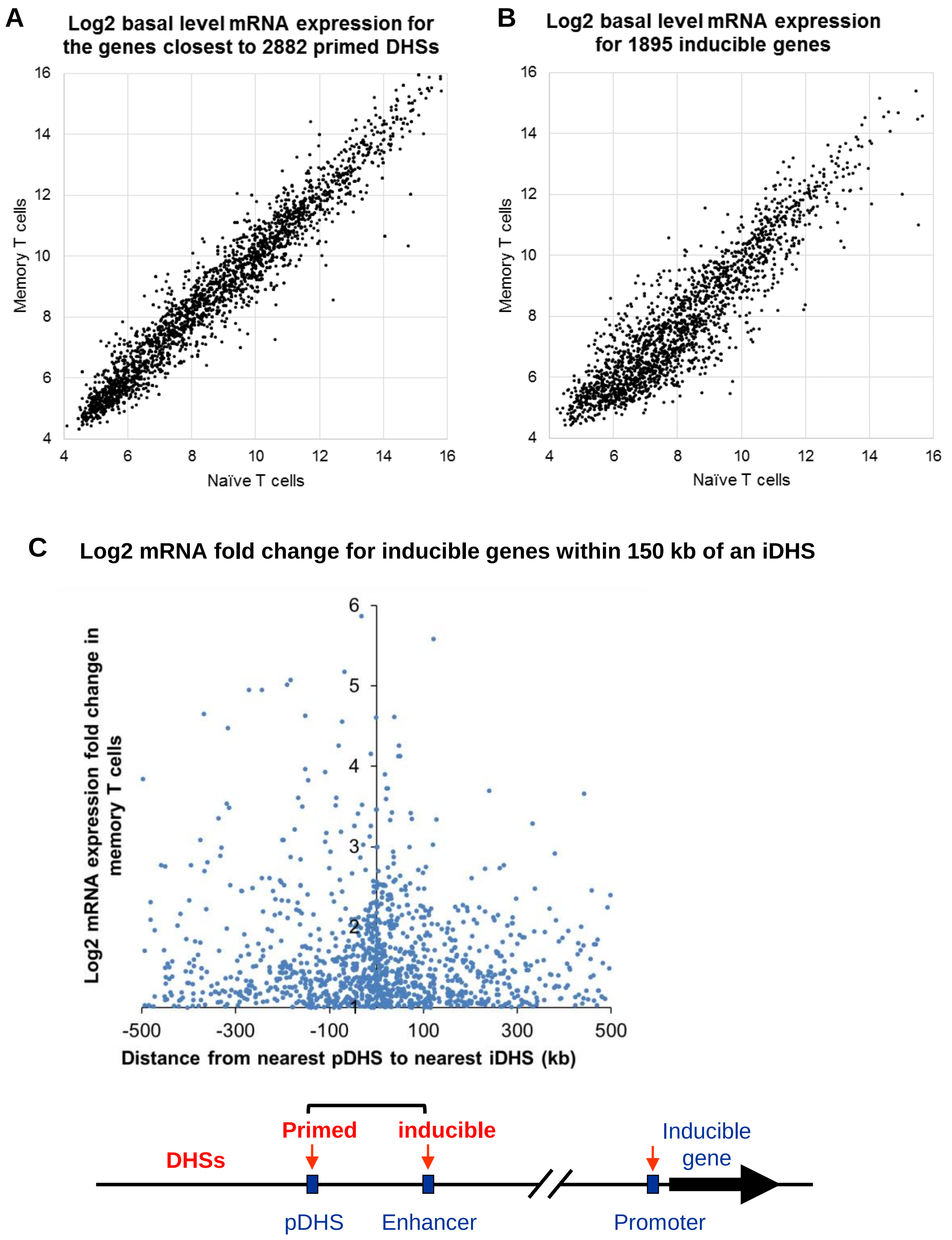

Primed DHSs reside close inducible DHSs which are linked to inducible genes.

Appendix Figure S3

Influence of primed DHSs on basal and inducible levels of gene expression.

Overlaps for TF ChIP peaks with pDHSs and iDHSs

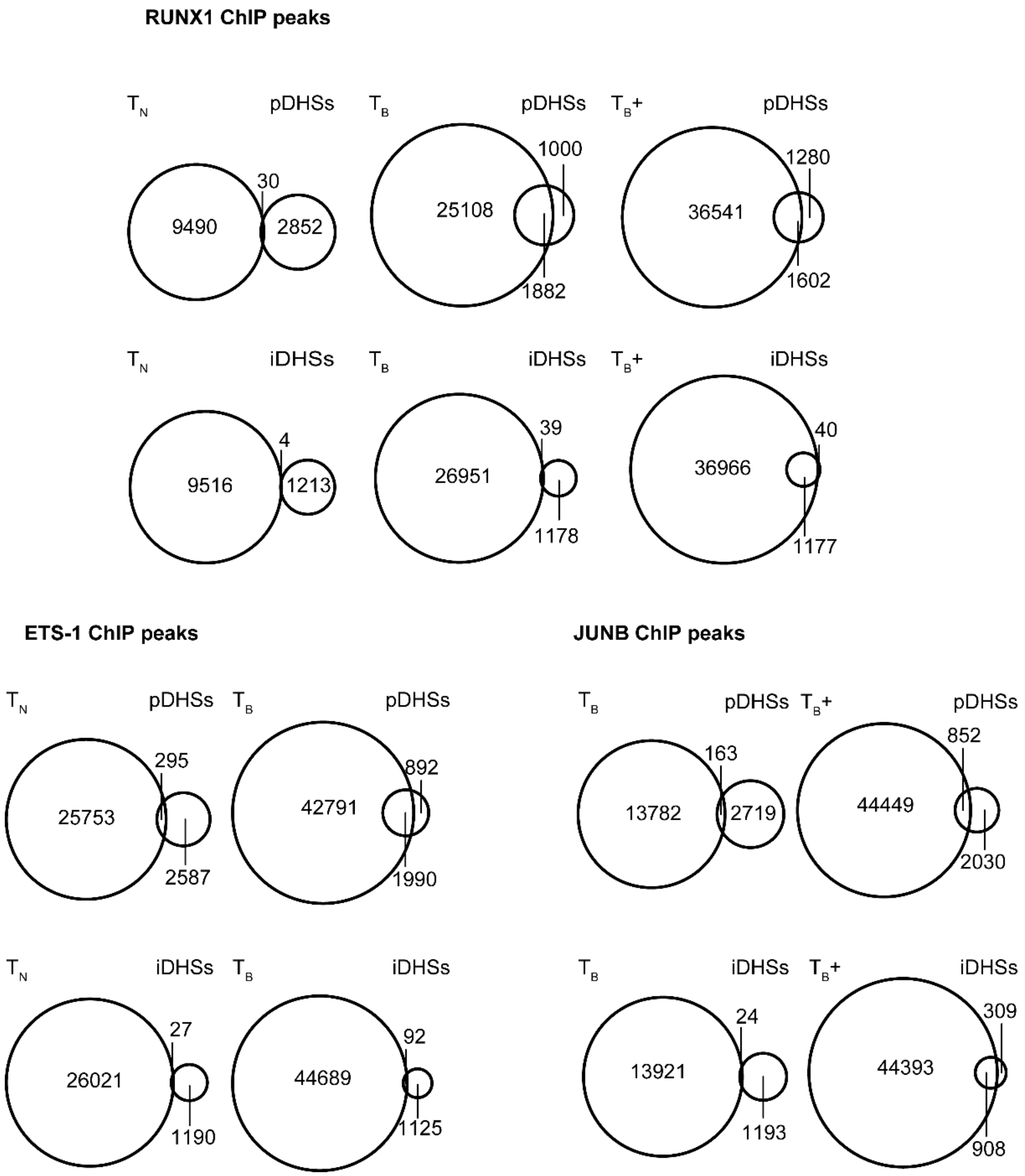

Appendix Figure S4

Overlaps for TF ChIP peaks with pDHSs and iDHSs.

A

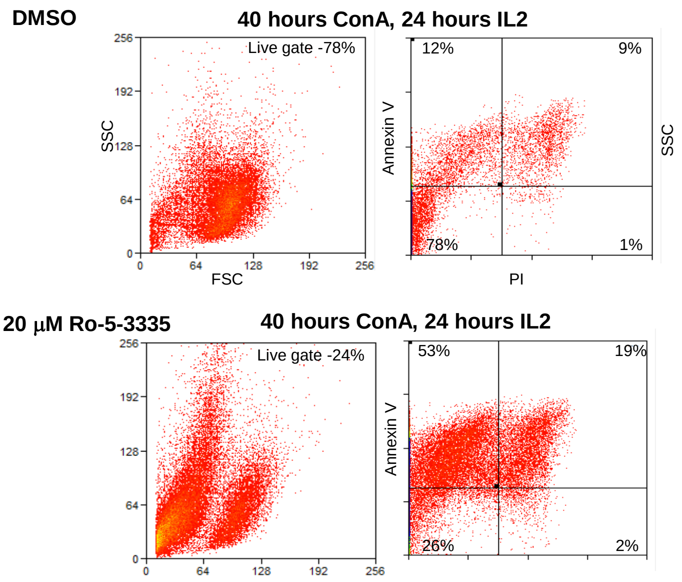

B

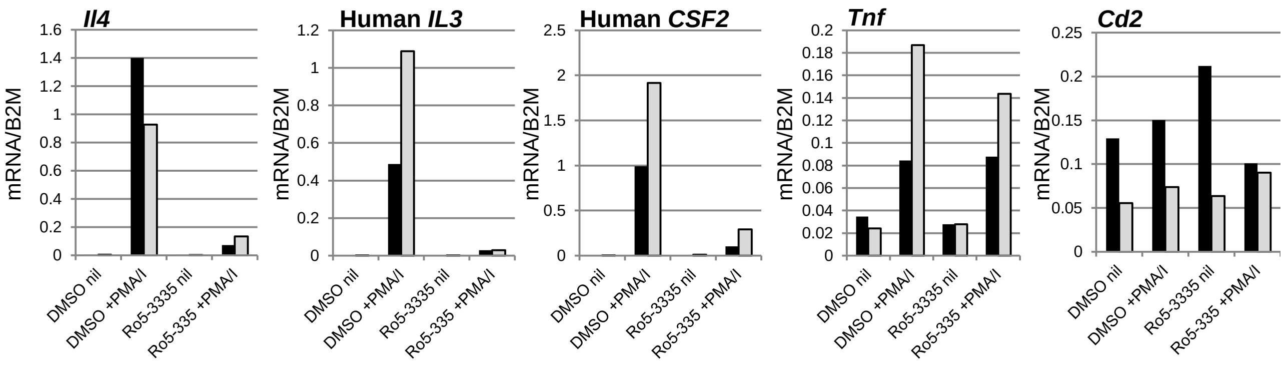

Appendix Figure S5

Suppression of inducible gene expression in memory-phenotype cells by an inhibitor of RUNX1 function.

A

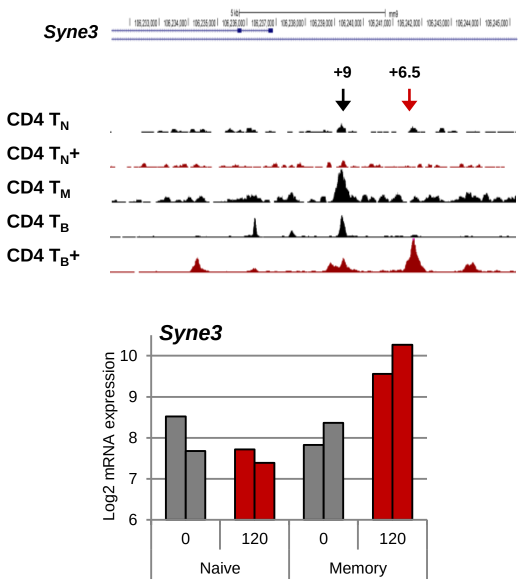

Syne3 +9Kb pDHS

CACGAAGTCATCCCTGATACACTGTCCCCCCCCAACCCACTGAGCTTCCCTGACTCCAGGCTACCACCCCCTGGG

NFAT

GTGGCCGATGTTTATGGCACCTTCTTACCCACCAAACTGTTTCCACAGAGCAGGGATGTGTCCCTGGCATCTTGC

TACCCATTCCAGCTCAGTCAAGGTCAAGTACATCGAAGGAATGCAGTAGTCCAGGGTATGTGGGTTACAGGGCGGA

RUNX

AGAGCCCAGATCCCAGCCACAAACACGTGTCTCTCTGGCTTCCAGACCACAGCCAGCTCCTGCTAGGAGCTTCAT

RUNX ETS ETS

GACTAATCTTGCCACTTCCTGTGGGTGACAAACACAGGAACTGAGGGCCATCCTCAGTCCTCGTCACTAAAGCCA

AGAACTCACCCAGGGATAGTCAGCCTGGACACCTGGGTGATCTTACACAGAGAAGCGGGAATAAGGGAGGCCTCC

3x constitutive: ETS/RUNX, ETS, RUNX
1x inducible: NFAT

Syne3 +6.5Kb iDHS

GGCTGGAAAGCCTTTTGTCTAAGTCATAATTATAACCACCTCTCTGTGGGCTGCCAGTAAGCTGTCAAGATGAATG

EGR NFAT/AP-1

AGCAACAGGCGGGCGTGGGGGTATGACCAGAATTGAGTTCTGGAAACCATCGATCAAGTATCAATAGCAAAGGA

CAAGAGAACAGGAAACGGAAATCAGAATCCCAGGGTGGAACTTCTAGGGGGTGCTCTCTACCAGATGTGGGAGGA

ETS

ATAGCCCCGGAAGAGCAGCTGGCAGAAGTAACTGCTTGCCACAGGTAGAGAGGGGAGGAAAGTCAGAACAGATT

TCGGGGTTTTGTGGAACCCTAAAGCAACCTGGGGGCAGATAGGCGGGCAGACCCAACACTTAGGGAAAGGGAGG

1x constitutive: ETS,
2x inducible: NFAT/AP-1, EGR

B

Rad50 +50Kb pDHS

ETS

TGAAAGCCAAAGCTTCCTGTTAAACCTGAACACCATGCCTGCCCTAGCTACCTCAGCTCTCAGGAGACAGGACTA

ETS

ACAAAGTCACTCCAGTGGTAGGAAGGCAAGTTTCACAGCTATCACCATGCAAAGGATGTGCGCGGACTCCCTTCC

GATA

ATTTGCTGGCCTCTTATCTGATATATACAAAAAAAGAAACTAAACGTAATGCCGCTGCCTCTGTACAATCATTCCGT

AP-1

GATCTTCTGGTCACCAGCCAAAGAGAAAGAAATGACTCAACATAAATACGTCTAAGTATCAGATGCAGGCTGCTGA

AACAGTAAGCCTGGAGAAGCCTCTCTGCTTCACCAGGTCTCCATTTTCTAACCCACGCTCATCTCTGCCTGATACT

Rad50 +52Kb pDHS

ETS RUNX

ATGTCCTTAGGAGCACTGGCTGCCTTGCAAACTCTCTGGCCAGGAAGACTCAGATCATGTGGTCTGCCCAGATGC

NFAT/AP-1 GATA

AGCCTGGATGGCCCACCCCCGCCTGGGCCCAGCTCTTTTCTAAGAGTTTCCTTTGGGTACTTTTCTTATCACACT

GATA

TGGGCCTATCCCTATCTGCGCTTTCACAGAACCCATTATGTCATCAGCTAAATGTGTCTGCTGCAGCCAATATGA

GATA

GTCGTACAGGGCTTTGGAAGAGAAACTGCATTTTCATTTTGTTGTAAGATGTTGCAAAGATAAGAATTCTTAGAA

ETS

AACTCTCAAAGACTTGCTCAGGAAGGGGTTTGAGAAGGCAAAGCTGGCAACTAAGGTCATACAGGAGAGGCTGT

Rad50 +56Kb iDHS

NFAT/AP-1

TGCTACTGATGCAGCAGAAAGTCTTCTAGGAAAACTTAAAACATCAACCAGAGCCCAGCTCGCTCCTCTGCCCAG

ACTCCCAGACTTCTTTCAAAGTCTCAAGGTAGTTCGGGCAGACAACAGGCAGTGAGGGGAATAACACTACCTTCT

ETS AP-1

GAGGAGCTGGGTCTACCTAGAATTCTTCCTCCCCCAGTCACTAGATGCCTCAGTGCAGTGACACACAGGGCCTG

ETS

AGTGGGTGTATGGGGACATTTTTGCTCATCCTCAGCATGCTGAGGTATCGGCTATGAGTGAGATGCAACAAGGGT

C

Cxcr3 -3Kb pDHS

CAAGGTCAGAGGCAACTACCTAGCCAAAAGCTCACAGCCTGTGTTCTGGTCTCTGGGCCCTCCCTGCTCTGCAG

ETS AP-1 T-box

CAGAGAGGGCTGAGGAGGAAGTTAGTCAGAGTGAAGCTGCCTGGGGCCTCATCAGTGGTCAAGGTGTGAAATG

Sp1 T-box RUNX

TGCACCTGGCTCTTCTCTGCCTCCCCTCCCCCTGTTGTTTTTCATTCTTTCACACCTCTGAGCTGTGGTTTGGGG

ETS

AGACAGGAGGCTGGGAGATAGACACCTTTGTGCCCAGACCCTCACAGGAAGGAGGGCCCCCTCATCAGGAACA

ETS NFAT

GGGGTCATTTCCTTCTCTCTCCCAGCCTCTGCTTCACAGCCACACAATAGCCTCCCTAGTCATGTTTCCAAAAGC

Cxcr3 +3Kb iDHS

GATA AP-1

AGATCTGCAGCCTCAGATAAGACCCAGCCTGTCAGGGTGAGTCATGGCATGTTGGTGACTTCATTGGGAGAAAGT

NFAT

GACAGTGCAGGGGGTAGCCCTGTCACCTACCCTTCTGGAAAGCACTGGTTATTTTTTCCACTCAGCTTTAGACATT

GAAACTGCTCCTTGTTGCCCACTTCTTGCTTTGGTGGCTTCATGCTAATGTCTGCTAGCAAAGAAATCATCAGGCC

Appendix Figure S6

Composition of pDHSs and iDHSs

Supplementary Methods

Mice

C42 transgenic mice containing a 130 kb AgeI genomic DNA fragment of the human *IL3/CSF2* locus were described previously (Mirabella et al., 2010). These mice were back-crossed onto C57 Black 6 mice (C57BL/6J) for greater than ten generations, and the genotype confirmed as essentially all C57BL/6J, before breeding the transgene back to homozygosity.

Cell culture, purification and stimulation

CD4 or CD8 single positive T cells were isolated using MACS CD4 (L3T4) or CD8a (Ly2) microbeads. $CD4^+$ and $CD8^+$ single positive naïve T cells (T_N) were isolated from the spleens of C42 transgenic mice using MACS $CD4^+$ and $CD8^+$ T cell Isolation kits followed by enrichment for naive cells using MACS CD62L+ microbeads according to the manufacturer's instructions (Miltenyi Biotec, Auburn, CA). CD4 positive memory T cells (T_M) were purified from splenocytes using the Easy Sep mouse memory $CD4^+$ T cell Isolation Kit according to the manufacturer's instructions (Stem Cell Technologies). Actively proliferating T blast cells (T_B) were generated from purified quiescent CD4 and CD8 positive splenic cells by culturing at $5x10^5$ cells/ml in IMDM with 2μg/ml Concanavalin A for 40 hours to activate TCR signaling pathways. Proliferating cells were then maintained at $5x10^5$ cells/ml in 50 U/ml recombinant mouse IL-2 (Peprotech) for an additional 2-3 days before harvesting. Cells were stimulated with 20 ng/ml phorbol myristate acetate (PMA) and 2 μM Calcium Ionophore A23187 (CaI) in IMDM supplemented with 50 U/ml IL-2 at a concentration of $1x10^6$ cells/ml for up to 4 hours. Jurkat T cells were cultured in RPMI supplemented with 10% fetal bovine serum.

ConA Time course

$CD4^+$ T_N cells were cultured in IMDM at $5x10^5$ cells/ml. Cells were treated with Concanavalin A for 0, 3, 8 and 16 hours before harvesting for RNA extraction, reverse transcription and qPCR analysis of gene expression.

Flow cytometry (FACS)

$CD4^+$ T_M, $CD4^+$ T_N and $CD4^+$ T_B cells were labeled with CD4 PerCP-Cy5.5 (BD Biosciences 561115), B220 eFluor 450 (e-biosciences 48-0452-82), CD44 PE-Cy7 (BD Biosciences 560569), CD62L APC (e-bioscience 17-0621-81), CD3 FITC (e-biosciences 11-0251-83) and CD25 PE (e-biosciences 12-0251-83) before analysis on the CyAn ADP analyser (Beckman Coulter).

DNase I hypersensitive site analysis

DNase I digestions were performed as previously described, by adding DNase I in the presence of NP40 to intact cells and digesting for just 3 min (Bert et al., 2007). A range of DNase I concentrations were used to achieve the optimal digestion, with the amount varying depending on the cell type and the concentration of cells in the reaction. Briefly, permeabilized cells were digested in DNase I buffer (60 mM KCl, 15 mM NaCl, 5 mM $MgCl_2$, 100 mM Tris-HCl pH 7.4, 1 mM EGTA pH 7.4, 0.3 M sucrose, 0.2% NP40, 1 mM $CaCl_2$ and DNase I) at $1.5x10^6$ cells/ml for T_M and $1.5x10^7$ cells/ml for T_N and T_B. Digestions were carried out at 22°C for 3 minutes and SDS was then added to a final concentration of 0.5% to terminate the reaction. Cell lysates were treated with 0.5 mg/ml Proteinase K at 37°C overnight followed by 0.2 mg/ml RNase A at 37°C for 1 hour. DNA was purified by phenol/chloroform extraction.

For DNase-Seq, samples were first analyzed by qPCR to identify the optimally digested samples. The ratio of PCR signal was determined between active regions which were expected to be DNase I hypersensitive and inactive regions which should not be cleaved. Optimally digested samples gave a ratio of ~0.4 to 0.7 for active/inactive. DNA fragments (~80-300 bp) were isolated from favorable samples by agarose gel extraction and further validated by qPCR analysis before library preparation. Primer pairs used to validate samples were, for the active region, TBP promoter 5' – TGCAGTCAAGAGCGCAACTG; and 5' – CACCGCTACCGGACTCGAT; and for the inactive region, a gene desert on chr 1 5'-TGCTCCACAGTGTCCATGTACA; and 5'-AGCAATTTCATGGGTGAGAGAAG.

Southern blot DNA hybridization analysis of human T cells was performed as previously described (Baxter et al., 2012; Mirabella et al., 2010), using either (i) BamHI and the previously used probe for the human *IL3* -34 kb DHS (Baxter et al., 2012), or (ii) using EcoRI and a PCR-amplified segment of the transgene prepared using the primers AGGTGGGTGATTCTGTTGATG and TGGTCCAGCTTATCATGCAG for the human *CSF2* +30 kb DHS region.

Chromatin Immunoprecipitation

For the antibodies H3K27ac (ab4729) and H3K4me2 (Millipore 07-030), cells were crosslinked in PBS at 1x10^6 cells/ml with 1% formaldehyde (~0.34 M) for 10 minutes at room temperature with rotation. For the antibodies, JUNB (sc-46), RUNX1 (ab23980), ETS1 (sc-350), and BRD4 (Bethyl laboratories), cells were re-suspended in PBS at 3.3x10^6 cells/ml and cross-linked with 0.83 mg/ml Di(N-succinimidyl) glutarate (DSG - Sigma) for 45 minutes at room temperature with rotation. Cells were washed four times with PBS and further crosslinked in PBS at 2x10^6 cells/ml with 1% formaldehyde (~0.34 M) for 10 minutes at room temperature. All crosslinking reactions were quenched by adding 4 volumes of PBS and 0.125 M glycine, the cells were washed twice in PBS and incubated with Buffer A (10 mM HEPES pH 8.0, 10 mM EDTA, 0.5 mM EGTA, 0.25% Triton X-100) at 4°C for 10 minutes followed by Buffer B (10 mM HEPES pH 8.0, 200 mM NaCl, 1 mM EDTA, 0.5 mM EGTA, 0.01% Triton X-100, 0.25% SDS) at 4°C for 10 minutes. Crosslinked chromatin was sonicated using a Biorupter^{TM} (Diagenode) in IP buffer I (25 mM Tris 1 M, pH 8.0, 150 mM NaCl, 2 mM EDTA, pH 8.0, 1% TritonX-100 and 0.25 % SDS) at a concentration of 2x10^7 cells/ml to give fragments of an average length of ~500 bp. The supernatant was diluted with 2 volumes of IP buffer II (25 mM Tris, pH 8.0, 150 mM NaCl, 2 mM EDTA, pH 8.0, 1% TritonX-100, 7.5 % glycerol) and immunoprecipitation was performed for 2 hours at 4°C using 1-2μg of antibody coupled to 15 μl protein G Dynabeads (Dynal) per 2x10^6 cells. Beads were washed with Buffer 1 (20 mM Tris-HCl, pH 8.0, 150 mM NaCl, 2 mM EDTA, pH 8.0, 1 % TritonX-100, 0.1 % SDS), twice with Buffer 2 (20 mM Tris-HCl pH 8.0, 500 mM NaCl, 2 mM EDTA, pH 8.0, 1 % TritonX100, 0.1 % SDS), LiCl buffer (10 mM Tris-HCl, pH 8.0, 250 mM LiCl, 1 mM EDTA, pH 8.0, 0.5 % NP40, 0.5 % Na-deoxycholate) and twice with TE/NaCl buffer (10 mM Tris-HCl pH 8.0, 50 mM NaCl, 1 mM EDTA). DNA and protein complexes was eluted in 100 μl elution buffer (100 mM NaHCO3, 1% SDS) and the crosslinks were reversed by incubating with proteinase K at 65 °C overnight. DNA was extracted using Agencourt AMPure (Beckman Coulter) beads according to the manufacturer's instructions and analyzed by qPCR using SYBR Green reagents on the StepOnePlus (Applied Biosystems).

Luciferase assays

Assays of the GM-CSF promoter plasmids were carried out using the previously described luciferase reporter gene plasmids pGM and pGM-GME which contain the vector pXPG plus the -624 to +28 bp region of the human *CSF2* (GM-CSF) promoter alone (pGM) or with the -3 kb *CSF2* enhancer upstream of the promoter (pGM-GME) (Bert et al., 2000; Cockerill et al., 1999). Additional plasmids were constructed from pGM plus the indicated DNA fragments upstream of the *CSF2* promoter. The +28 and +30 kb DHSs downstream of *CSF2*

were first sub-cloned by amplifying the entire +28 to +30 kb region using PCR primers to introduce a BglII site upstream and a KpnI site downstream of the DHSs. The upstream BglII primer sequence TTTGTTGAGATCTTACAGGATTTCTT contains a natural BglII site, and the downstream primer sequence TTGGCTCTGGTACCCACAACAACACCA was used to introduce a KpnI site into the sequence TGGTGTTGTTGTGGGGACCAGAGCCAA. This +28/30 BglII-KpnI segment was cloned into the BglII and KpnI sites of pGM. Additional sub-clones were prepared by either (i) deleting a BamHI segment containing the +28 kb DHS to create pGM+30, or (ii) deleting a SalI segment containing the +30 kb DHS to create pGM+28. The minor +22 kb DHS downstream of *CSF2* was sub-cloned as an XhoI-digested DNA fragment into the XhoI and SmaI sites of pGM after using the PCR primers TACCAATCCTCGAGGAAGCAGGTT and GGGCAGACAATTTGTCCCGCATGG to amplify a 1.6 kb region associated with a peak of H3K4me2 in blast cells which encompassed 2 clusters of highly conserved DNA sequences.

The co-transfected control Renilla luciferase plasmid pGME-RL was derived from pGM-GME by replacing the firefly luciferase gene with the Renilla luciferase gene from the pTK-RL vector (Promega). For each assay $4.5x10^6$ Jurkat T cells were transfected by electroporation using 5 μg of the test plasmid and 1μg of pGME-RL. Cells were cultured for 24 hours before stimulation with 2 μg/ml PMA and 2 μM calcium Ionophore for 8 hours before harvesting for analysis. Assays were performed using the Promega Dual-Reporter Luciferase Assay System kit according to the manufacturer's instructions.

Assays of the *IL3* promoter plasmids were carried out as above but using the previously described luciferase reporter gene plasmid pIL3H (Hawwari et al., 2002), which contains the vector pXPG plus the -559 to +50 bp region of the human *IL3* promoter, co-transfected with the control plasmid pRL-TK (Promega). These assays included previously described plasmids containing the human *IL3* -34, -37 or -41 kb DHSs cloned upstream of the IL-3 promoter in pIL3H (Baxter et al., 2012). The plasmid pIL3H-4.1/-1.5 includes both the -4.1 and -1.5 kb *IL3* DHSs, and was made inserting the entire -4.3 to -0.17 kb region upstream of the human *IL3* promoter as a BamHI-ScaI fragment in between the upstream HindIII site and the -0.17 kb ScaI site within the *IL3* promoter of pIL3H, by performing a partial ScaI/HindIII digestion of pIL3H. Assays of *Ccl1* gene elements were performed as above in the context of the *IL3* promoter. Mouse *Ccl1* locus DNA segments were amplified from Black 6 mouse DNA by PCR, followed by restriction enzyme digestion and cloning into appropriate sites upstream of the *IL3* promoter in pIL3H. PCR amplification used the primers CTGGATCCACCCCTAAAACTGG and GGAACCAAAACCTGCAGTTGCT for cloning the Ccl1 -3.7 kb region into the BglII and SacI sites, and TCACATCATGAAGATCTCTTGTTCTTCTG and GATTCAGAAAGGCTAGTGTTAGCACC for cloning the *Ccl1* -35 kb region into the BamHI site. One of the -35 kb region primers has a mismatch (underlined) to create a BglII site. The -35 kb PCR product was digested with BglII and BamHI to give compatible ends for cloning into the pIL3H BamHI site.

Gene expression analyses by RT-qPCR

Total RNA was isolated using TRIzol™ (Invitrogen) according to the manufacturer's instructions. Contaminating DNA was removed using the Turbo DNA-free™ kit (Ambion) and first strand cDNA synthesis was carried out using M-MLV Reverse Transcriptase (Invitrogen) according to the manufacturer's recommendations. Gene expression was analyzed by qPCR using SYBR Green reagents and run on the StepOnePlus (Applied Biosystems). The mouse genome PCR primers used were as follows:

B2m: 5'-TTCTGGTGCTTGTCTCACTG 5'-CAGTATGTTCGGCTTCCCATTC
Jun: 5'-GCCGGAAAAGGAAGCTGGAGCGG 5'-CTGTTCCCTGAGCATGTTGGCCG
Fos: 5'-TCCAAGCGGAGACAGATCAAC 5'-TTTTTCCTTCTCTTTCAGCAGATTG
Junb: 5'-TCACGACGACTCTTACGCAG 5'-CCTTGAGACCCCGATAGGGA
Jund: 5'-GAGGCTGATCATCCAGTCCAA 5'-CCTCGCTAGCTGCCACCTT

Fosb: 5'-CGAGAAGAGACACTTACCCCA 5'-GTTTCCGCCTGAAGTCGATCT
Nfatc1: 5'-TGGAAGCAAAGACTGACCGG 5'-CTGGTTGCGGAAAGGTGGTAT
Nfatc2: 5'-GGTACCACCTACTTGGCCCAA 5'-TGGGAGGGATGCAGTCACAG
Il4: 5'-ACAGGAGAAGGGACGCCAT 5'-ACCTTGGAAGCCCTACAGA
Il10: 5'-CCTGGGTGAGAAGCTGAAGACC 5'-CTTCACCTGCTCCACTGCCTTG
Tnf: 5'-CACGTCGTAGCAAACCACCAAGTGGA 5'-TGGGAGTAGACAAGGTACAACCC
Cd2: 5'-GCACAAATGGGATGACTAGGCTGG 5'-GCACAGGTCAGGGTTGTGTTG
Ccl1: 5'-CCAGACATTCGGCGGTTG and CAGCAGCAGGCACATCAG

Human genome primers:

IL-3: 5'-GGACTTCAACAACCTCAATGGG 5'-TTGAATGCCTCCAGGTTTGG
GM-CSF: 5'-CACTGCTGCTGAGATGAATGAAA 5'-GTCTGTAGGCAGGTCGGCTC
GAPDH: 5'-CCTGGCCAAGGTCATCCAT 5'-AGGGGCCATCCACAGTCTT

mRNA microarray analysis and clustering

Total RNA was isolated using Trizol™ (Invitrogen) according to the manufacturer's instructions. RNA quality was determined using an RNA 6000 Pico Chip run on the Bioanalyzer 2100 system (Agilent). 100 ng of RNA was labeled with Cyanine 3-CTP using the One-Color Microarray-Based Gene Expression Analysis protocol (Low Input Quick Amp Labeling) according to manufacturer's instructions (Agilent). Labeled RNA samples were purified using RNeasy mini spin columns (Qiagen) and the concentration of cRNA was determined using a Nanodrop spectrophotometer. 600 ng of each cRNA was hybridized at 65°C overnight to the SurePrint G3 Mouse GE 8x60K Microarray kit v1 design ID 028005 (Agilent) following the Agilent hybridization protocol. After washing, microarrays were scanned on an Agilent G2565C Microarray Scanner using the Profile AgilentG3_GX_1Color for 8x60K microarrays (Dye channel: Green; Scan region: Scan Area (61 x 21.6 mm); Scan resolution (µm): 3; Tiff: 20 bit). Probe signals were extracted via the Agilent Feature Extraction software (version 10.7.1.1), protocol GE1_107_Sep09, using grid number 028005_D_GeneList_20131202 (SurePrint G3 Mouse GE 8x60K). Arrays were normalized via quantile normalization in *R* using the *limma* package (Smyth et al., 2005). Transcript annotations were aggregated into unique gene names. The mean value was computed for genes with more than one transcript. To identify common trends in differentially regulated genes, the top 1% of genes showing the highest variance of expression among datasets was selected and a Pearson correlation matrix was computed for these genes in R. Clustering of correlations was carried out using *cluster 3.0* (de Hoon et al., 2004), with -e 2 -g 2 -m mas parameters. T_M-specific genes were defined as those with a $CD4^+$ T_N +PMA/I over $CD4^+$ T_N gene expression fold change < Log_2 1 and a T_M +PMA/I over T_M expression fold change > Log_2 1 (1895 genes). Boxplots of gene expression fold changes were plotted using *R*.

CRISPR deletion of the IL3 -34 kb DHS, and analyses of mutated cells

Cloning of single cells after transfection:

Plasmids were constructed using the pSpCas9(BB)-2A-GFP vector (PX458-Addgene). Single guide RNAs (sgRNA) were designed using the online CRISPR design tool (http://tools.genome-engineering.org). Guides were chosen to have minimum off-target activity as computationally predicted by the design tool. Guides were designed to the 5' and 3' end of the DNase I hypersensitive site located 34 kb upstream of the IL-3 gene. 34 kb 5' CACCGTGAATTCCAGCAATGTTCG 5' Reverse complement: AAACCGAACATTGCTGGAATTCAC 34 kb 3': CACCGATGAGCACCCTTCGGGCTC 3' Reverse complement: AAACGAGCCCGAAGGGTGCTCATC.

100 μM of the sense and antisense oligonucleotides were annealed and phosphorylated with 5U T4 Polynucleotide kinase at 37°C for 30 min followed by 95°C for 5 min before cooling slowly to room temperature. The annealed oligonucleotides were ligated into the PX458 vector using the Golden Gate assembly cloning strategy where digestion and ligation occur in the same reaction. PX458 (100 ng) was mixed with 1 μM of the annealed oligonucleotides, 20 U BbsI, 10 mM ATP, 5 μg BSA and 750 U T4 DNA ligase. Reactions were incubated at 37°C for 5 minutes and 20°C for 5 minutes for 20 cycles followed by 1 cycle at 80°C for 20 minutes. 1 μl of the ligation mix was transformed into DH5α E.coli. Plasmids were prepared from positive clones and the DNA sequenced to check for the insertion.

Transfection of CRISPR reagents:

$2x10^6$ Jurkat T cells were resuspended in 300 μl antibiotic free RPMI supplemented with 10% fetal bovine serum and transfected with either 5 μg of PX458 (WT) or 5 μg of the plasmids PX458 containing the 5' and 3' sgRNAs ($-34^{-/-}$). Cells were electroporated at 240 V, 950 μF and transferred to a 6 well plate in a final volume of 5 ml antibiotic free RPMI supplemented with 10% fetal bovine serum. 48 hrs post transfection, GFP positive cells were sorted into 96 well plates after which clones were expanded for approximately 14 days before screening for deletions by PCR using the primers -34 kb F: CCCTCCCTTGGTAACTCCTTGTTG -34 kb R: ATCACAGTCCAGGGATGTTGGTCC, followed by agarose gel electrophoresis and ethidium bromide staining. Single cell cloning was performed by culturing in 40% Jurkat T cell-conditioned media.

DNase I accessibility assay:

The $-34^{-/-}$ clones, WT clones and untransfected Jurkat T cells were stimulated for 3 hours with PMA/I before harvesting and digestion with a range of DNase I concentrations as described previously. DNA was recovered and analyzed for the level of digestion by qPCR analysis using the following primers TBP Pr: 5'-CTGGCGGAAGTGACATTATCAA and 5'-GCCAGCGGAAGCGAAGTTA Chr18: 5'-ACTCCCCTTTCATGCTTCTG and 5'-AGGTCCCAGGACATATCCATT IL3 -37 DHS 5'- CAGGTCTTTCTCAAACAACCTCCC and 5'-CATGCCTGGGGATGAGATGACAC

Gene expression:

The $-34^{-/-}$ clones, WT clones and untransfected Jurkat T cells were stimulated with PMA/I for the times indicated. RNA was extracted and reverse transcribed to cDNA as previously described. Analyses of human *IL3* and *JUN* expression used the following primers:
IL-3: 5'-GGACTTCAACAACCTCAATGGG and 5'-TTGAATGCCTCCAGGTTTGG
JUN 5'- GTTTGCAACTGCTGCGTTAG and 5'-CAGGTGGCACAGCTTAAACA
GAPDH 5'-CCCACTCCTCCACCTTTGAC 5'- ACCCTGTTGCTGTAGCCAAAT

RUNX1 inhibitor studies

$CD4^+$ T cells were purified using MACs CD4 (L3T3) microbeads according to the manufacturer's instructions (Miltenyi Biotec, Auburn, CA). Cells were cultured at $5x10^5$ cells/ml in IMDM with 2 μg/ml Concanavalin A and either 20 μM CBFβ-Runx1 Inhibitor II, Ro5-3335 (CAS 30195-30-3 – Calbiochem) or an equivalent volume of DMSO. This inhibitor is reported to inhibit RUNX1 function, but has not been shown to interact directly with RUNX1 (Cunningham et al., 2012). After 40 hours the Concanavalin A was removed and the cells were maintained in IMDM at $5x10^5$ cells/ml with 20 μM Ro5-3335 or DMSO and 50 U/ml recombinant mouse IL-2 (Peprotech) for 24 hours.

The proportion of apoptotic and dead cells was determined using the PE Annexin V

Apoptosis detection kit I (559763) according to the manufacturer's instructions. Samples were analyzed on the CyAn ADP 8 analyser (Beckman Coulter). Dead cells were removed using the MACs dead cell removal kit (Miltenyi 130-090-101) according to the manufacturer's instructions prior to stimulation for 2 hours with PMA/I and subsequent RNA extraction and gene expression analyses.

Data analysis

Alignment, peak detection and generation of coverage tracks of DNaseI-Seq and ChIP-Seq datasets

The high throughput DNA sequence data presented in this paper is a compilation of the following data sets generated using either Illumina or SOLiD DNA sequencing technology:

Cell type	Experiment	Total reads	Aligned reads	Peaks	Sequencing method
Naïve CD4	DHS biorep1	38309802	10203874	23708	SOLiD
Naïve CD4	DHS biorep2	58330158	46938886	19231	Illumina
Naïve CD4+PMA	DHS	40377875	32713929	13896	Illumina
Naïve CD4	H3K4me2	91115520	74261046	53728	Illumina
Naïve CD4	Ets1	134062111	62938297	26048	Illumina
Naïve CD4	Runx1	63099439	54391910	9520	Illumina
Naïve CD8	DHS	39631761	10378873	32599	SOLiD
T-Blast CD4	DHS biorep1	364257736	190745253	62430	SOLiD/Illumina
T-Blast CD4	DHS biorep2	52712326	40420460	36798	Illumina
T-Blast CD4	H3K4me2	257985861	85122187	49567	SOLiD/Illumina
T-Blast CD4	Ets1	148169018	103284815	44781	Illumina
T-Blast CD4	H3K27Ac	66001536	57001599	24357	Illumina
T-Blast CD4	Runx1	79060600	59829702	26990	Illumina
T-Blast CD4	JunB	112830344	53757476	13945	Illumina
T-Blast CD4	BRD4	38054961	30823748	28063	Illumina
T-Blast CD8	DHS	196310782	44891153	75724	SOLiD
T-Blast+PMA CD4	DHS	176497317	145118657	62018	Illumina
T-Blast+PMA CD4	Runx1	39010278	31240495	28143	Illumina
T-Blast+PMA CD4	JunB	48380238	37298812	45301	Illumina
T-Blast+PMA CD4	H3K4me2	67439153	59018996	24640	Illumina
T-Blast+PMA CD4	H3K27Ac	51931052	42625817	31357	Illumina
T-Blast+PMA CD4	BRD4	45643715	36928604	35839	Illumina
Memory CD4	DHS	257065491	190457076	60794	Illumina
Memory+PMA CD4	DHS	217398014	149031692	63041	Illumina

DNA sequence reads were aligned to the July 2007 mouse genome data from the Build 37 assembly by NCBI and the Mouse Genome Sequencing Consortium (mm9) using the *bowtie* aligner (Langmead et al., 2009) with --all --best --strata --v 2 -m 1 as parameters. For the SOLiD reads, the colorspace version of the mm9 genome was used, and reads were aligned as color fasta using the same parameters. For datasets split into several runs, and all datasets were aligned together using *bowtie*. In all cases, outputs were in the SAM format as instructed to *bowtie* using the -S switch, converted to BAM, sorted then indexed using the *view*, *sort* and *index* functions of the *samtools* package (Li et al., 2009). For the human

IL-3/GM-CSF transgene reads were aligned to a custom-built index of the human genome covering chromosome 5 of the March 2006 human genome data from the Build 36 assembly by NCBI and the Human Genome Sequencing Consortium (hg18), using the same parameters and methodology. This index was built using the *bowtie-build* function of the bowtie package, using the FASTA sequence of human chromosome 5 from the hg18 genome. For datasets requiring merging of Illumina and SOLiD outputs, sorted BAM files were merged using the *merge* function of the *samtools* package. Peak detection, tag extension and generation of coverage tracks was subsequently performed using the *macs14* package (Zhang et al., 2008). In all cases, peak detection was performed with bowtie's built in calculation of the maximum number of tags at one location based on a binomial distribution, using the --keep-dup=auto switch. For datasets spanning less than one lane, this switch was used to compute coverages using the -w and -S switches. In the case of datasets spanning several lanes, the --keep-dup=all switch was used to compute coverages. Genome sizes were adjusted depending on whether the reference genome was mouse or human, using the -g mm or -g hs switches, respectively.

Unions of data from CD4$^+$ T_N, T_B, T_B+ and T_M T cells

To compare sequence tag counts at two stages for common and specific regions, we generated the unions of the peaks. Due to the intrinsic imprecision of DNaseI fragment lengths, this can result in non-overlapping, redundant summit coordinates. To obtain non-redundant summit populations, pairwise unions were generated by concatenating, sorting then merging peak summits within 400 bp using *sort* and *merge* functions of the *bedtools* package (Quinlan and Hall, 2010). For T_N and T_M summits, a tag cut-off of 50 and 20 was used to discard ambiguous peaks. Tag counts were retrieved ±1 Kb around the union of summits from coverage files using the *annotatePeaks* function of the *Homer* package (Heinz et al., 2010) using -hist 10 -ghist -wig -size 2000 as parameters, summed up ±200 bp around the summit to retrieve tags corresponding to the length of a DHS, then subsequently ordered by Log_2 fold change, whereby totals of value 0 were replaced by 1 in order to eliminate infinite values. A BED file representing summits sorted by Log_2 fold change was created and corresponding DHS and ChIP tracks (e.g. T_M alongside the CD4$^+$ T_B/T_N fold change axis) were retrieved using annotatePeaks using the same parameters, however sorted according to the Log_2 fold change-sorted input BED file. Heatmap images were generated using *Java Treeview* (Saldanha, 2004).

Definition of pDHSs, iDHSs and average profile generation

To define the pDHSs, DHSs enriched more than 3-fold in the CD4$^+$ T_M over T_N union were selected (3054 DHSs). From the 3054 T_M-specific DHSs, regions which were also called as peaks in the CD4$^+$ T_B, were defined as the pDHSs (2882 DHSs). For the inducible sites, 6823 iDHSs were enriched more than 5.5-fold in the T_B+ over T_B union. The 1217 strongest iDHSs were enriched more than 11-fold. To compute average DHS and ChIP profiles ±2 Kb around the pDHSs or iDHSs, the *annotatePeaks* function of the *Homer* package was used with -size 4000 -hist 10 -wig as parameters.

Normalization of ChIP-Seq and DNase-Seq experiments

For genome coverage tracks, scales were adjusted to control genes by visual inspection (*TBP*, *ACTB*, *GAPDH*, enhancers of the *TCR α* and *β* for DHS and histone modification tracks, enhancers of the *TCR α* and *β* for Ets1, Runx1 and *Fli1-Ets1*, *Runx1* enhancers, respectively, and *Fos*, *Jun* promoters for JunB) to reflect signal to noise ratios rather than tag count normalization. For average profiles, scaling was performed by retrieving coverages in 10,000 randomly selected, 4000 bp wide regions in order to compute background levels, so that values of average profiles were scaled proportionally to the ratio of average background levels of all datasets with respect to CD4$^+$ T_B. For heatmaps, contrasts proportional to the numbers of reads divided by the number of lines in heatmaps were used.

Distance and gene expression analyses

pDHSs or iDHSs closest to the 1895 T_M-specific genes were retrieved using the bedtools slop function. A RefSeq annotation of TSSs from the mm9 genome was used as a BED file. The distance to the TSS was obtained using the -D switch, which calculates the distance of features relative to their orientation. Genes were grouped according to distance from the pDHS or iDHS to the TSS of the T_M specific genes in 25 kb intervals. The median fold change of gene expression in T_M+PMA/I compared to T_M was plotted relative to the distance. Genes were also grouped according to fold induction of expression in $T_{M.}$ The median distance to the closest iDHS and pDHS was calculated for each group. For the distance between iDHSs and pDHSs, the closest pDHS was determined as outlined above and the distances were calculated using the coordinates of the nearest iDHS. p-values were calculated via a χ^2 test using the random expectation value of each comparison as the expected value, vs the observed value. The empirical calculation using base-pair probabilities was performed as in the following example:

To calculate the random expectation value of 312 pDHSs out of 683 falling within [-25 Kb, +25 Kb] of 6,823 iDHS, the size of the base-space of iDHSs +/- 25 Kb was first computed: there were a total of 6,823 iDHSs thus corresponding to 6,823x2x25,000=341,150,000 bp. The size of the mm9 genome being 2,725,765,481 bp, the probability of encountering an element at random in this base-space is thus 341,150,000/2,725,765,481=0.125157502. The random expectation value of finding 312 elements out of 683 in this base-space is thus the number of these elements times the probability of encountering an element at random in this base-space or 683x0.125157502=85.48257421 pDHSs. Calculations were then performed using the reciprocal population as the empirical reference (e.g. using the base-space of 683 pDHSs as the reference and the probability of 6,823 iDHSs falling within [-25 Kb, +25 Kb] of those).

Since these calculations yield one point with no standard deviation (the expected intersections), a χ^2 test was used to calculate the p-value with k=1 degree of freedom. χ^2 was calculated as (Observed – Expected)²/Expected. P-values were computed by obtaining the corresponding value of the computed χ^2 value in the χ^2 probability density distribution (function *CHISQ.DIST.RT* in *Excel*). The following example depicts how the p-value of finding 312 pDHSs falling within [-25 Kb, +25 Kb] of 6,823 iDHS while the random expectation value was of 85.48257421:

χ^2=(Observed – Expected)²/Expected

χ^2=(312-85.48257421)²/312

χ^2= 600.2409809

p(χ^2)= *CHISQ.DIST.RT*(χ^2,k)= $\frac{1}{2^{\frac{k}{2}}\Gamma\left(\frac{k}{2}\right)}\, x^{\frac{k}{2}-1}e^{-\frac{x}{2}}$

where k=1 degree of freedom and $\Gamma(n) = (n-1)!.$

p(χ^2)= 1.4838E-132

Random-expectation values were checked by simulating the background model using bootstrapping, using 10,000 iterations of intersections of random coordinates (generated via *bedtools random* using the *system* command in *R*) of size equal to one group vs the other actual group and subsequently swapped. The generated values were comparable to the calculated random-expectation values (e.g. 85.6 with σ=11.8 for the above example, yielding a Z-score Z=(X-μ)/σ of 19.119, corresponding to a p-value of 1.67 E-80 using the *NORM.S.DIST* function in *Excel*).

For comparisons that involved classes of gene expression fold change and distance of iDHSs to the TSS of an inducible gene or distance between pDHSs to the nearest iDHS around inducible genes (Fig 4F, H), p-values were calculated via t-tests using similarly-sized populations of random DHSs. For classes of expression fold change versus distance of iDHSs to the TSS of an inducible gene (Fig 4F), we selected 6823 DHSs at random while using the same inducible genes. For classes of expression fold change versus distance between pDHSs to the nearest iDHS around inducible genes (Fig 4H), we selected 6823 DHSs overlapping with no pDHSs. T-tests were carried out on all distance values for each class of fold change, between values for pDHSs and values for random DHSs, using a two-tail distribution.

Motif discovery, heatmaps and tables

De novo motif discovery was performed using the *findMotifsGenome* function of the *Homer* package. For motif heatmaps, top-enriched position weight matrices (PWM) identified via de novo motif discovery were matched against regions spanning ±1 Kb around summits of previously defined and sorted unions, using the *annotatePeaks* function of the *Homer* package, with -size 2000 -m as parameters. For consistency, the same, non-redundant matrices were used in all comparisons, with the ones showing the lowest p-value across datasets retained where applicable. Heatmap images were generated via *Java Treeview*. For the motif tables, the non-redundant matrices were matched to regions spanning ±200 bp around summit unions and the total number of occurrences for each motif was calculated. Motifs were then split into two classes: constitutive (RUNX, ETS, KLF, GATA, E-box) and inducible (AP-1, NFAT, EGR, NF-κB, CREB/ATF)

Motif co-occurrence clustering

In order to estimate whether pairs of motifs co-localized significantly in a given population of sequences, an enrichment Z-score was computed between actual motif intersection counts against 1,000 randomly selected sequences from the merged transcriptionally active space of T-cells corresponding to the scope of study as a background. For the enrichment of co-localization of motifs in pDHSs and iDHSs, this corresponded to the unions of DHSs from naïve, blast, memory T-cells and blast, stimulated blast $CD4^+$ T-cells, respectively. A custom script performing motif annotation, intersection in actual populations as well as in randomly selected regions of the active space and enrichment calculation was written in the *R* statistical language. Background co-occurrence was thus estimated using bootstrapping (1,000 repetitions) of motif mapping and co-association counts (within 50bp) in randomly selected regions within the background, using equally sized populations as the original number of peaks (2882 for pDHSs and 1217 for iDHSs). Annotation of PWMs to sequences of pDHSs and iDHSs was performed using *Homer annotatePeaks*, using -size 400 -m -mbed as parameters. For the sake of consistency across datasets, built-in PWMs from *Homer* most closely related to PWMs enriched in de novo motif discovery were used. Resulting BED outputs were extended by 50bp using the *slop* function of the *bedtools* package. To obtain motif co-occurrence, extended motif BED files were all intersected using the *intersection_matrix* function of the *pybedtools* package (Dale et al., 2011). Enrichment was assessed by calculating a Z-score, which was represented by $Z=(X-\mu)/\sigma$, whereby μ and σ would represent background average co-occurrences and standard deviation of average, background co-occurrences. Z-scores were clustered via *cluster 3.0* using -e 2 -g 2 -m m as parameters. Heatmaps images were generated using *Java TreeView*.

Digital genomic footprinting

The $CD4^+$ T_B, T_B+ and T_M datasets had >140,000,000 mouse-aligned read counts and signal to noise ratios high enough to undergo digital genomic footprinting. For this purpose, the *wellington_footprints* function of the *Wellington* suite (Piper et al., 2013) was used with standard parameters (p-value score, threshold 10^{-20}) on BED3-converted peaks. Briefly,

Wellington computes scores for each possible position of a footprint and for a number of different footprint sizes. The scores reflect the enrichment of positive strand reads starting near the 5' end of a footprint and the enrichment of negative strand reads starting near the 3' end of a footprint. To retrieve DNaseI cuts in the DHSs, the *dnase_wig_tracks* function of the *Wellington* suite was used with standard parameters on BED3-converted peaks. To compute average cut profiles and matrices on foot-printed motifs, PWMs were first matched to foot-printed regions using the *annotatePeaks* function of the Homer package, using -size given -m -mbed and thus output as BED files. Average cut profiles and strand imbalance matrices were generated using the *dnase_average_profiles* and *dnase_to_javatreeview* functions of *Wellington* using standard parameters (sort by footprint occupancy score for matrices) on BED3-converted footprinted motif files. For heatmaps of matrices corresponding to strand imbalance, *Java TreeView* was used for the generation of images. A *Perl* script was written so as to perform these steps automatically for all motifs, using *Java Treeview* from the command line using -f -x -- -o -s as parameters, whereby a scaling factor was computed so that all heatmaps were of equal height (600 pixels), with width being constant. Footprint Occupancy Scores (FOSs) are computed as the ratio of cuts observed within a binding site to those immediately outside (Neph et al., 2012). To retrieve FOSs, a development *Python* script of the *Wellington* suite (*wellington_score_heatmap.py*) was used, which outputs FOSs per input interval in an exploitable text format. To compute significant differences in overall occupancy for AP-1 motifs, a paired t-test was performed using FOSs at AP-1 motifs in T_B and T_{B+}. The p-value was retrieved using the two-tailed T distribution.

For heatmaps of footprints in T_M cells containing ETS and RUNX motifs, TM footprints were first annotated with ETS and RUNX motifs using *bedtools annotatePeaks*, using -m -mbed as parameters. The resulting files were intersected with all T_M footprints to reflect the ones containing these motifs and converted to bedGraph files. The presence of these motif-containing footprints was assessed by using *bedtools annotatePeaks* using the T_M/T_N DHS union as input and the motif containing footprints as the bedGraph coverage files, with -hist 10 –ghist -bedGraph as parameters. Heatmap images were generated using *Java TreeView*.

Annotation of genomic features and gene ontology analyses

In order to determine the genomic location of the DHSs (intergenic, intragenic, promoter, exonic, etc.) the *annotatePeaks* function of the *Homer* package was used with default options, with outputs redirected to text files. Numbers of occurrences for each genomic feature type were derived using the *DCOUNTA* function in *Excel* and plotted as percentages of total DHSs as piecharts. For gene ontology analyses, *annotatePeaks* was used using the -go switch, returning the closest transcription start site (TSS) and performing gene ontology analysis based on these. For gene ontology analyses of expression microarray results, the TSS of genes was used as input.

Supplementary References

Baxter, E.W., Mirabella, F., Bowers, S.R., James, S.R., Bonavita, A.M., Bertrand, E., Strogantsev, R., Hawwari, A., Bert, A.G., Gonzalez de Arce, A., *et al.* (2012). The inducible tissue-specific expression of the human IL-3/GM-CSF locus is controlled by a complex array of developmentally regulated enhancers. J Immunol *189*, 4459-4469.

Bert, A.G., Burrows, J., Osborne, C.S., and Cockerill, P.N. (2000). Generation of an improved luciferase reporter gene plasmid that employs a novel mechanism for high-copy replication. Plasmid *44*, 173-182.

Bert, A.G., Johnson, B.V., Baxter, E.W., and Cockerill, P.N. (2007). A modular enhancer is differentially regulated by GATA and NFAT elements that direct different tissue-specific patterns of nucleosome positioning and inducible chromatin remodeling. Mol Cell Biol *27*, 2870-2885.

Cockerill, P.N., Bert, A.G., Roberts, D., and Vadas, M.A. (1999). The human granulocyte-macrophage colony-stimulating factor gene is autonomously regulated in vivo by an inducible tissue-specific enhancer. Proc Natl Acad Sci U S A *96*, 15097-15102.
Cunningham, L., Finckbeiner, S., Hyde, R.K., Southall, N., Marugan, J., Yedavalli, V.R., Dehdashti, S.J., Reinhold, W.C., Alemu, L., Zhao, L.*, et al.* (2012). Identification of benzodiazepine Ro5-3335 as an inhibitor of CBF leukemia through quantitative high throughput screen against RUNX1-CBFbeta interaction. Proc Natl Acad Sci U S A *109*, 14592-14597.
Dale, R.K., Pedersen, B.S., and Quinlan, A.R. (2011). Pybedtools: a flexible Python library for manipulating genomic datasets and annotations. Bioinformatics *27*, 3423-3424.
de Hoon, M.J., Imoto, S., Nolan, J., and Miyano, S. (2004). Open source clustering software. Bioinformatics *20*, 1453-1454.
Hawwari, A., Burrows, J., Vadas, M.A., and Cockerill, P.N. (2002). The human IL-3 locus is regulated cooperatively by two NFAT-dependent enhancers that have distinct tissue-specific activities. J Immunol *169*, 1876-1886.
Heinz, S., Benner, C., Spann, N., Bertolino, E., Lin, Y.C., Laslo, P., Cheng, J.X., Murre, C., Singh, H., and Glass, C.K. (2010). Simple combinations of lineage-determining transcription factors prime cis-regulatory elements required for macrophage and B cell identities. Molecular cell *38*, 576-589.
Langmead, B., Trapnell, C., Pop, M., and Salzberg, S.L. (2009). Ultrafast and memory-efficient alignment of short DNA sequences to the human genome. Genome Biol *10*, R25.
Li, H., Handsaker, B., Wysoker, A., Fennell, T., Ruan, J., Homer, N., Marth, G., Abecasis, G., and Durbin, R. (2009). The Sequence Alignment/Map format and SAMtools. Bioinformatics *25*, 2078-2079.
Mirabella, F., Baxter, E.W., Boissinot, M., James, S.R., and Cockerill, P.N. (2010). The human IL-3/granulocyte-macrophage colony-stimulating factor locus is epigenetically silent in immature thymocytes and is progressively activated during T cell development. J Immunol *184*, 3043-3054.
Neph, S., Vierstra, J., Stergachis, A.B., Reynolds, A.P., Haugen, E., Vernot, B., Thurman, R.E., John, S., Sandstrom, R., Johnson, A.K.*, et al.* (2012). An expansive human regulatory lexicon encoded in transcription factor footprints. Nature *489*, 83-90.
Piper, J., Elze, M.C., Cauchy, P., Cockerill, P.N., Bonifer, C., and Ott, S. (2013). Wellington: a novel method for the accurate identification of digital genomic footprints from DNase-seq data. Nucleic Acids Res *41*, e201.
Quinlan, A.R., and Hall, I.M. (2010). BEDTools: a flexible suite of utilities for comparing genomic features. Bioinformatics *26*, 841-842.
Saldanha, A.J. (2004). Java Treeview--extensible visualization of microarray data. Bioinformatics *20*, 3246-3248.
Smyth, G.K., Michaud, J., and Scott, H.S. (2005). Use of within-array replicate spots for assessing differential expression in microarray experiments. Bioinformatics *21*, 2067-2075.
Zhang, Y., Liu, T., Meyer, C.A., Eeckhoute, J., Johnson, D.S., Bernstein, B.E., Nusbaum, C., Myers, R.M., Brown, M., Li, W.*, et al.* (2008). Model-based analysis of ChIP-Seq (MACS). Genome Biol *9*, R137.
