# Supplementary material for: Inducible chromatin priming is associated with the establishment of immunological memory in T cells
Source: EMBO J. 2016 Jan 21;35(5):515–35. doi: 10.15252/embj.201592534 (PMC4772849; doi:10.15252/embj.201592534)
Supplement: Supplementary file 2 — Expanded View Figures PDF [file EMBJ-35-515-s002.pdf]

Expanded View Figures

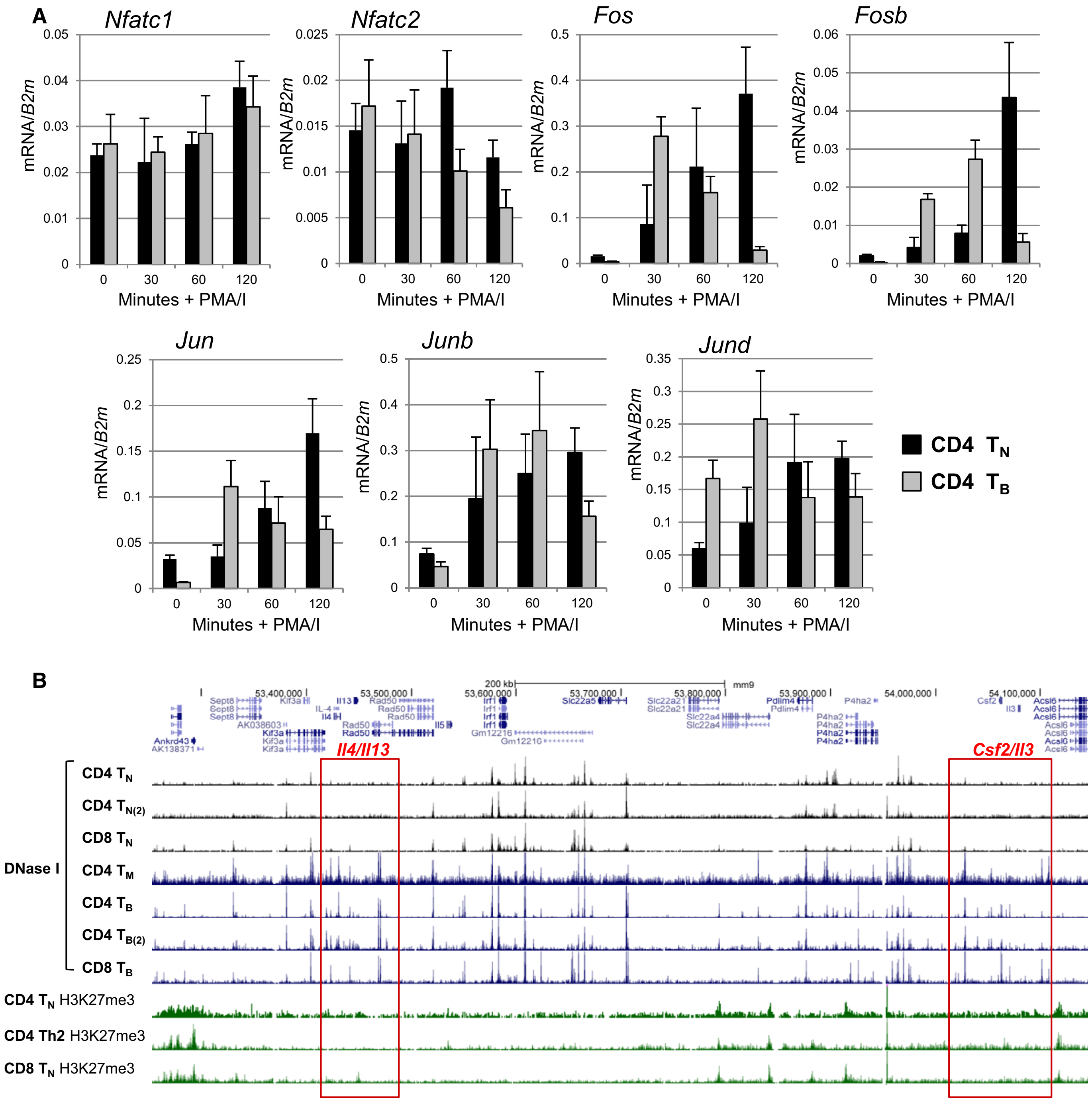

Figure EV1. Comparisons of mouse T_N and T_B TF mRNA and chromatin profiles.

A PCR analyses of mRNA expression of NFAT and AP-1 family transcription factors in CD4 T_N and T_B stimulated with PMA/I for the times indicated. Expression levels are normalized to the levels of *B2m*. Values represent the mean and SEM of 3 to 10 replicates, with a median of 5 replicates for each value shown.

B UCSC genome browser shot of a 900-kb region of mouse chr11 showing DNase I-Seq for CD4 and CD8 T_N, T_B, and T_M plus published datasets for H3K27me3 in CD4 and CD8 T_N and Th2 cells. T_N(2) and T_B(2) represent biological replicates.

 © 2016 The Authors

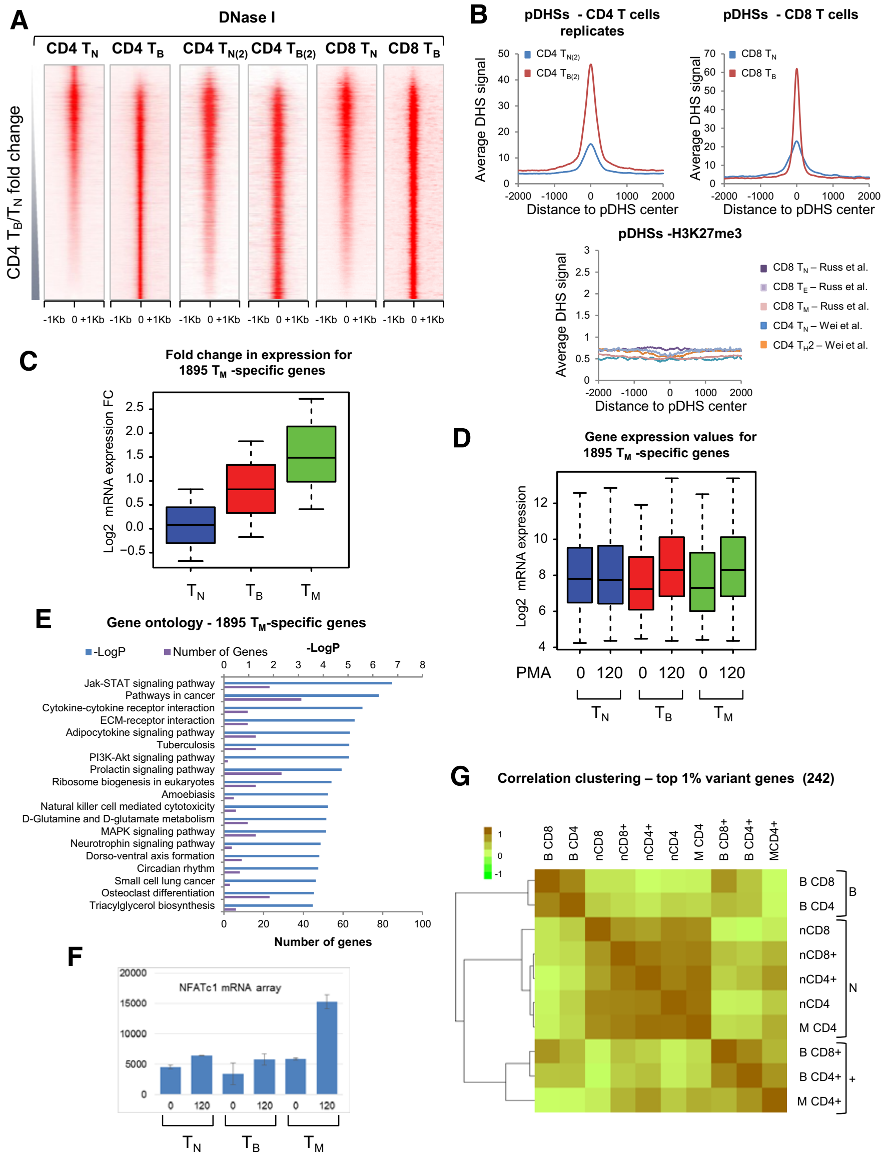

Figure EV2. CD4 and CD8 T cells share a common set of pDHSs.

A Density maps representing the DNase-Seq peaks in the cell types indicated at the top, shown in the order of increasing DNase-Seq tag count signal for the CD4 T_B compared to CD4 T_N. $T_B(2)$ and $T_N(2)$ are biological replicates.

B Average DNase I profile at the pDHSs in CD4 $T_B(2)$ and $T_N(2)$, CD8 T_B and T_N, plus the average H3K27me3 profiles for publically available CD4 and CD8 datasets in T_N T_M, effector T cells (T_E), and Th2 cells.

C, D Boxplots of log2 mRNA expression fold change (C) and absolute expression levels (D) of the 1,895 T_M-specific genes in CD4 T_N, CD4 T_B and CD4 T_M. Boxes represent the first and third quartile, respectively. Bottom and top whiskers represent the first and third quartile minus and plus 1.5 times the interquartile range.

E Gene ontology for the 1,895 T_M-specific genes.

F Mean cumulative mRNA array values for two alternatively spliced forms of the gene encoding NFATc1. Values are based on 4 separate micro-array values and are shown with SD.

G Hierarchical correlation clustering of mRNA levels for the top 1% of genes with the highest variance of mRNA expression between populations of CD4 and CD8 T_N, T_B, and T_M. Treatment with PMA/I is indicated by a "+" sign. Pearson correlations are shown according to the color scale (top left). B, N, and + (right) indicate the dominant groups resulting from clustering.

© 2016 The Authors

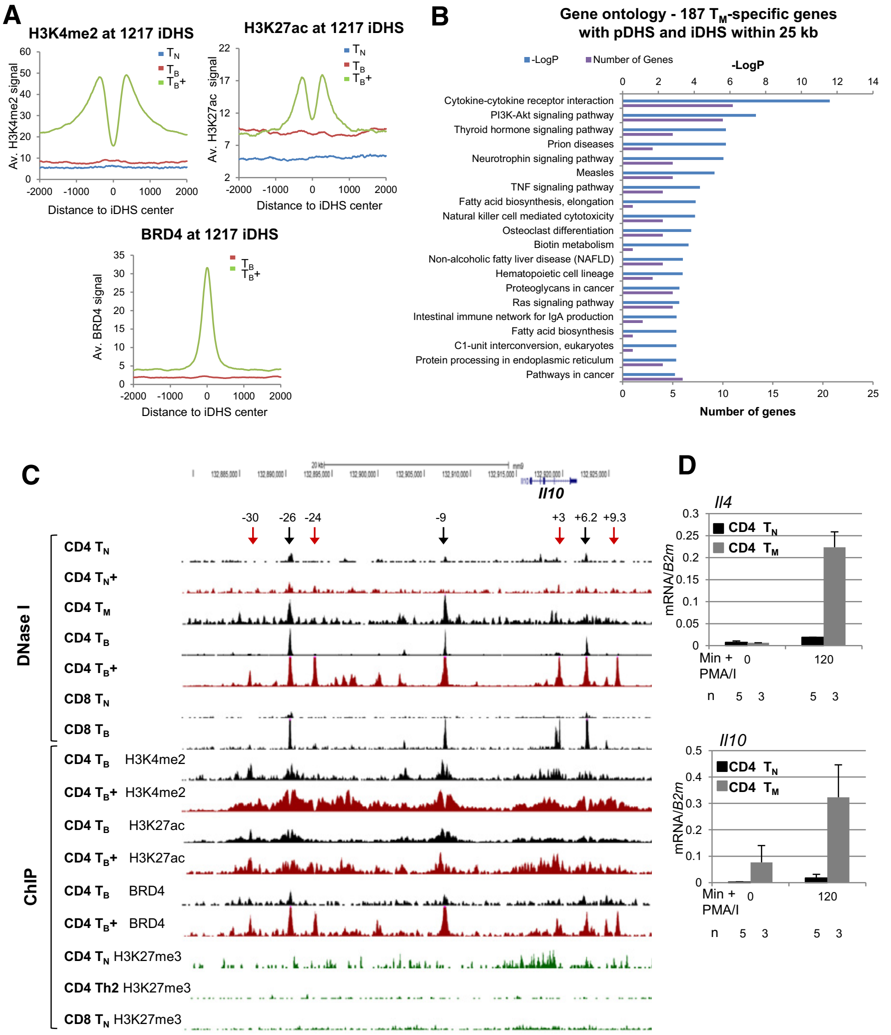

Figure EV3. Properties in inducible DHSs.

A Average H3K4me2, H3K27ac, and BRD4 signals at the 1,217 iDHSs in CD4 T_B and T_B^+, plus H3K4me2 and H3K27ac for T_N.

B Gene ontology for the 187 T_M-specific genes located within 25 kb of both a pDHS and an iDHS.

C DNase I-Seq and ChIP-Seq at the *Il10* locus in CD4 T_N, T_M, T_B, T_B^+, and Th2 cells and CD8 T_N and T_B.

D *Il4* and *Il10* mRNA expression in CD4 T_N and CD4 T_M stimulated with PMA/I for the times indicated. Relative mRNA values are expressed as in Fig 1C, with SEM. The number of replicates for each (*n*) is shown underneath.

 © 2016 The Authors

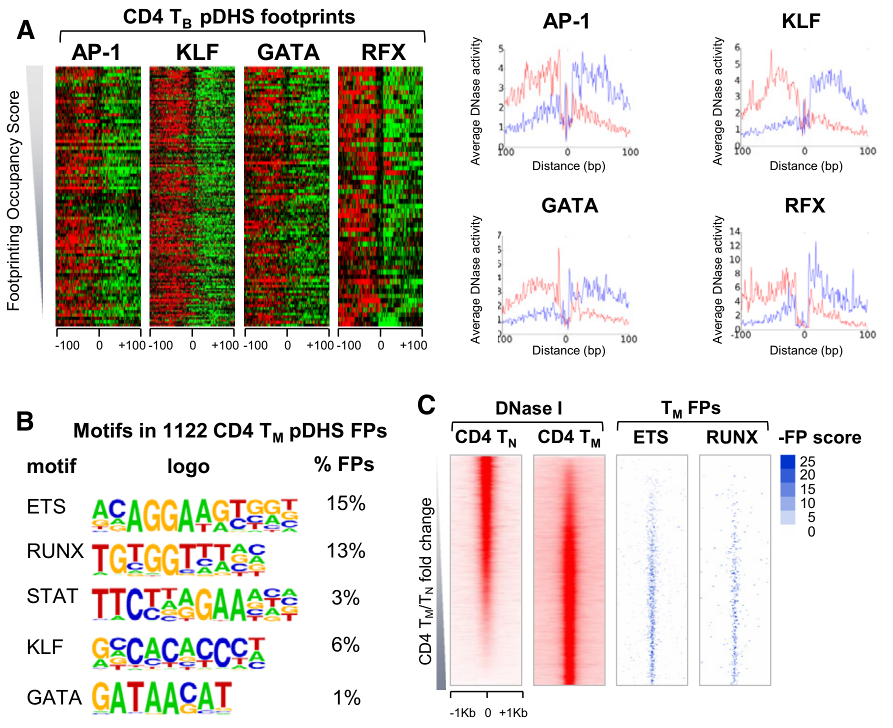

Figure EV4. DNase I footprints of TFs in T_B.

A Left: DNase I cleavage strand imbalance patterns within the footprints identified by Wellington at the pDHSs centered on the motif named at the top and ordered according to increasing FP occupancy score. Relative levels of DNase I cuts are shown within a 200-bp window with upper strand DNA cuts shown in red and lower strand cuts in green. Right: Average profiles of the DNase I cuts at the different motifs within the pDHSs, upper strand DNA cuts shown in red and lower strand cuts in blue.

B Results of the HOMER *de novo* motif search of pDHS digitally footprinted regions in CD4 T_M.

C ETS and RUNX motif-containing footprints (right) within DHSs in T_M sorted by T_M/T_N fold change (left).

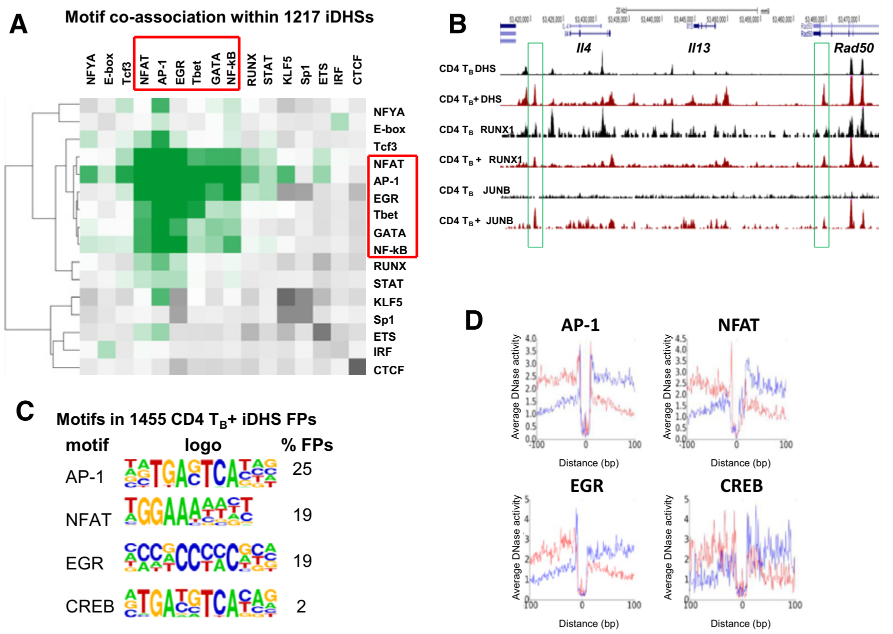

Figure EV5. Co-association of inducible TFs in iDHSs.

A Hierarchical clustering of motif co-association enrichments in iDHSs. Z-scores represent enrichment of observed versus background co-associations computed in 1,000 randomly selected, chromatin-accessible regions. Z-score scale as in Fig 6A.

B UCSC genome browser shot of the *Il4*, *Il13*, and *Rad50* loci showing DNase-Seq and ChIP-Seq for T_B and T_B^+.

C *De novo* motifs identified by HOMER within iDHS digital FPs.

D Average profiles of DNase I cuts at motifs within the iDHSs. Upper strand DNA cuts are shown in red and lower strand cuts in blue.

© 2016 The Authors
