# Supplementary material for: Inducible chromatin priming is associated with the establishment of immunological memory in T cells
Source: EMBO J. 2016 Jan 21;35(5):515–35. doi: 10.15252/embj.201592534 (PMC4772849; doi:10.15252/embj.201592534)
Supplement: Supplementary file 7 — Dataset EV5 [file EMBJ-35-515-s007.zip › EMBOJ_92534_DatasetEV5/Legend for Dataset EV5.docx]

**Legend for Dataset EV5**

Motif files for transcription factor consensus binding sequences identified in HOMER de novo motif-finding searches of iDHSs and pDHSs which include the 5 inducible motifs (AP-1, NFAT, EGR, NF-κB and CREB/ATF) and 5 constitutive motifs (ETS, RUNX, KLF, GATA, E-box) used for compiling the total numbers of motifs present in the specific DHSs in T_M_, T_B_ and T_B_+ as presented in Figures 9A and 9B. Also included are the IRF and STAT motifs identified in pDHSs and the composite NFAT/AP-1 motif identified in the iDHSs.
